# Strategies for ear elevation and the treatment of relevant complications in autologous cartilage microtia reconstruction

**DOI:** 10.1038/s41598-022-17007-3

**Published:** 2022-08-08

**Authors:** Zhicheng Xu, Yiyuan Li, Datao Li, Ruhong Zhang, Qun Zhang, Feng Xu, Xia Chen

**Affiliations:** Department of Plastic and Reconstructive Surgery, Shanghai 9th People’s Hospital, Shanghai Jiao Tong University School of Medicine, 639 Zhi Zao Ju Road, Shanghai, 200011 People’s Republic of China

**Keywords:** Outcomes research, Therapeutics

## Abstract

Despite various surgical techniques for ear elevation in autogenous cartilage microtia reconstruction, it is still challenging for plastic surgeons to obtain a satisfactory depth of the cephaloauricular sulcus and stable projection of the reconstructed ear. Here, the authors demonstrate individualized options for surgical approaches and relevant details for complication management. Between January 2014 and June 2020, a series of 895 patients who underwent the second stage of microtia reconstruction were reviewed. Complications occurred in 103 patients aged between 8 and 34 years. Recommended surgical selections, as well as appropriate strategies for complication prophylaxis and treatment, were shown to minimize the negative influence on the contour of the cephaloauricular sulcus according to individual conditions. We found that 78% of the patients were satisfied with the auricle contour with harmonious integrity. Individualized strategies for ear elevation and complication treatment contribute to symmetry and satisfactory projection of the reconstructed auricle.

## Introduction

Multiple modifications have been made in the first stage of autogenous cartilage microtia reconstruction^1–7^. From skin incision and flap dissection to cartilage fabrication and postoperative care, each procedure has gradually become standardized, which is helpful to achieve favorable results. However, it is comparatively difficult to acquire satisfactory cephaloauricular sulci after ear elevation despite varied selectable options for support materials, fascial flaps and skin grafts. Moreover, relevant complications, especially hypertrophic scarring, would impact the symmetry of the proposed projection. In this study, the authors introduce practical approaches and effective strategies for ear elevation and complication management.

## Methods

### Patients and methods

A series of 895 microtia patients underwent reconstructed ear elevation using modified Brent and Nagata’s techniques^6^ from January 2014 to June 2020. Among them, 539 cases were right sided, 335 cases were left sided, and 21 cases were bilateral. A total of 556 patients were male, and 339 were female (Table 1). Complications occurred in 103 patients aged between 8 and 34 years (Table 2). The presentation, prevention and treatment process of complications are shown in Tables 3 and 4. Factors/surgical techniques associated with risks of complications are reported in Table 5.

**Table 1:** Clinical data of the 895 microtia patients

Characteristic	No. of patients (%)
**Gender**	
Male	556 (62.1)
Female	339 (37.9)
**Age (years)**	
6–10	221 (24.7)
11–15	337 (37.7)
16–20	195 (21.8)
21–52	142 (15.8)
**Affected side**	
Right	539 (60.2)
Left	335 (37.5)
Bilateral	21 (2.3)
**Microtia type**	
Lobule	658 (73.5)
Concha	237 (26.5)
**Support materials**	
Single cartilage block	617 (68.9)
Combined cartilage block	92 (10.3)
EH composite wedge	186 (20.8)
**Donor site of skin graft**	
Scalp	763 (85.3)
Groin	132 (14.7)

**Table 2:** Complications after ear elevation in autologous cartilage microtia reconstruction.

Complication	Number	Percentage (%)
Partial skin necrosis	29	3.2
Facial flap necrosis/cartilage exposure	13	1.4
Infection	5	0.6
Exposure of support materials	4	0.5
Hypertrophic scar	52	5.8
Total	103	11.5

**Table 3:**
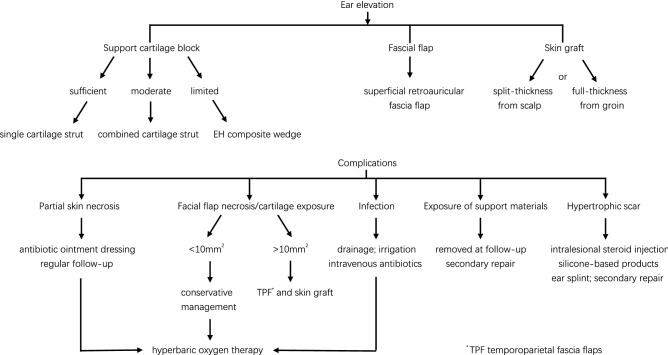
The algorithm of ear elevation and the treatment of relevant complications.

*TPF* temporoparietal fascia flaps.

**Table 4:** Predilection site/type, presentation, prevention and management of complications after ear elevation.

	Predilection site/type	Presentation	Prevention	Management
Partial skin necrosis	Rear margin of the auricle	Clearly-defined dark aera; fascia exposure	Meticulous hemostasis; full drainage; smooth dressing materials; symmetrically located bolster sutures; RFF^a^ harvest at posterosuperior region	Antibiotic ointment dressing; regular follow-up; HBOT^b^
Facial flap necrosis/ cartilage exposure	Rear margin of the auricle	Clearly-defined dark aera; cartilage exposure	Smooth surface at the rear of the framework; proper bolster pressure; RFF harvest at posterosuperior region	Antibiotic ointment dressing; regular follow-up; HBOT debridement; TPF^c^ + skin graft
Infection	EH^d^ application	Erythema; swelling; abscess; incision dehiscence; purulent secretion	Sterile operation; prophylactic antibiotics	Sufficient drainage; irrigation intravenous antibiotics; HBOT
Support materials exposure	EH application	Infection; fracture; exposure	Sterile operation; firmly fixation; proper sleeping position; traumatic force prevention	EH removal; secondary reconstruction
Hypertrophic scar	Mastoid region	Redness; itching; stiffness, scar contractures	Prevention of skin tension and wound complication; intralesional steroid injection; silicone-based products; ear splint	Intralesional steroid injection; silicone-based products; ear splint; surgical correction

^a^*RFF* retroauricular fascia flap, ^b^*HBOT* hyperbaric oxygen therapy, ^c^*TPF* temporoparietal fascia flaps, ^d^*EH* epoxide acrylate malelic and hydroxyapatite.

**Table 5:** Factors/surgical techniques associated with risks of complications.

Complication	Risk factors/surgical techniques
Partial skin necrosis	Careless hemostasis; insufficient drainage; unevenly distributed bolster pressure; improper harvest region of fascial flap
Facial flap necrosis/ cartilage exposure	Sharp protrusion at the rear of cartilage framework; overcompression of bolster; improper harvest region of fascial flap
Infection	Incomplete preventive sterile measures and prophylactic antibiotics, especially in EH application patients
Support materials exposure	Facial flap necrosis; infection; unstable fixation; improper sleeping position; traumatic force
Hypertrophic scar	Personal or family history of hypertrophic scarring; high skin tension; wound complications; not timely follow-up incomplete sequences of prophylaxis and treatment

This study was approved by the Ethics Committee of Shanghai Ninth People’s Hospital affiliated with the Shanghai Jiao Tong University School of Medicine (reference no. 2016-135-T84). All methods were carried out in accordance with the relevant guidelines and regulations. Informed consent was obtained from all participants and their legal guardians. Consent to publish from the participants and legal guardians of the minor participants for the mentioned case report was obtained.

### Selection of support materials for ear elevation

**Figure 1:**
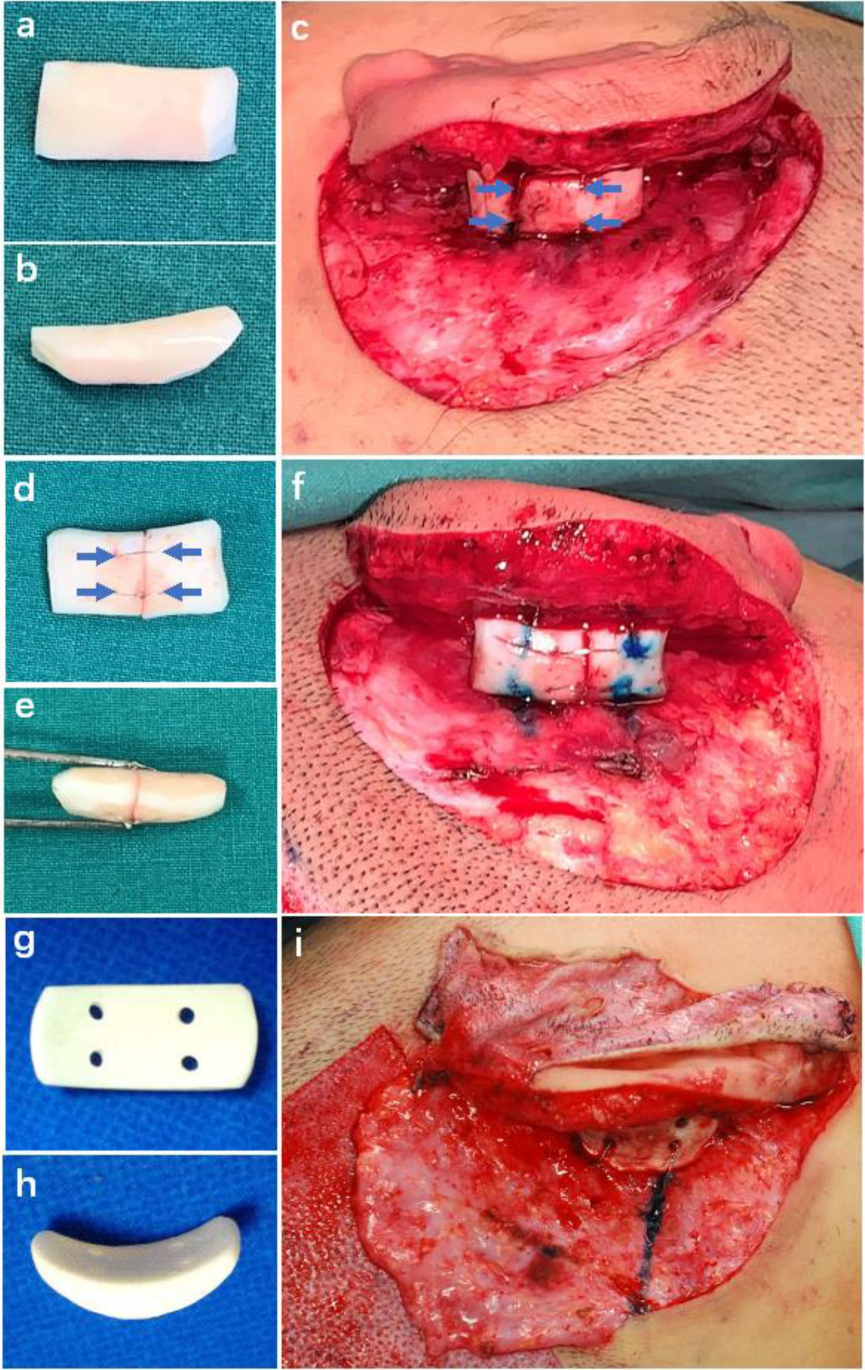
Schematic of support materials. (**a**) Anterior view of a single cartilage block. (**b**) Lateral view before plantation. (**c**) The cartilage block is fixed by stainless steel wires approximately 0.25 mm in diameter at four points. Two parallel wires go through the cartilage block and rear cartilage of the ear framework and then tie respectively, and the direction of the end of the steel wires remains internal with no tactile extrusion. The lower two correspondent points make the block fixed steadily at the base tissue. Blue arrows show the four fixation points. (**d**) Anterior view of combined cartilage blocks. Blue arrows show the connection points. (**e**) Lateral view before plantation. (**f**) The combined cartilage blocks were fixed steadily at four points. (**g**) Schematic of the EH (mixture of epoxide acrylate malelic and hydroxyapatite) composite wedge. Four evenly distributed pores are for steel wire fixation. (**h**) Lateral view of the EH composite wedge. The length was 20 mm; the height was 12 mm, and the thickness was 2 mm. (**i**) The EH composite wedge was fixed steadily at four points, with a split-thickness skin graft from the scalp and a nearly harvested superficial retroauricular fascia flap.

In the first stage, residual cartilage blocks were generally embedded subcutaneously in the chest wall as support materials for ear elevation. We find that the cephaloauricular angle can be well produced with a whole cartilage block if its length × height × thickness is at least 20 to 30 mm × 12 to 15 mm × 4 to 5 mm. It is commonly set at the middle third rear of the elevated auricular framework, adjusted to a similar projection compared with the contralateral normal side and then fixed by steel wires at four points (Fig. 1a–c). When the length of the residual cartilage is 10 to 20 mm, two such blocks may be connected by two steel wires and used as one support cartilage block (Fig. 1d–f). If there is not sufficient cartilage left, a special EH (a mixture of epoxide acrylate malelic and hydroxyapatite) composite wedge, which is 20 mm × 12 mm × 2 mm in size, would be an alternative substitute for cartilage strut (Fig. 1g–i).

### Surgical procedure

Ear elevation was performed approximately 6 months after the first stage. Skin incision was 5 mm from the margin of the auricle. The auricle was elevated to the anterior edge above the fascial plane. The selected support material was then firmly fixed at the posterior wall of the concha by steel wires. Afterward, a 7–8 cm × 4–5 cm superficial retroauricular fascia flap (RFF) and a 7–8 cm × 4–5 cm × 0.3 cm split-thickness skin graft from the scalp or full-thickness flap from the ilioinguinal region were covered successively. A tie-over dressing was applied for 9 to 10 days after the surgery.

### Postoperative complications

#### Partial skin necrosis

Solely partial skin necrosis with complete fascia is generally found at the rear margin of the auricle, which results from hematoma, unevenly distributed bolster pressure and the unequal blood supply of the facial flap. Meticulous hemostasis and full drainage are helpful to lessen hematoma. Meanwhile, smooth dressing materials and symmetrically located bolster sutures can distribute pressure evenly at the curve surface. Moreover, RFF at the posterosuperior region is recommended because it ensures adequate blood supply even at the edge^8^. Local skin necrosis can be well treated by some conservative treatments, including antibiotic ointment dressing, regular follow-up and HBOT^9^.

### Facial flap necrosis and cartilage exposure

Facial flap necrosis, commonly at the distal part, is definitely accompanied by subsequent cartilage exposure. The reasons include sharp protrusion at the rear of the cartilage framework, overcompression of the bolster and inconstant blood supply of the random facial flap. Thus, we think it important to fabricate a smooth framework with no sharp protrusion at the rear during the first stage. Suitable tightness of the bolster and harvest of RFF in a region with reliable vascular supply are also crucial to prevent facial flap necrosis. Conservative therapy strategies as described above are effective for exposure areas less than 10 mm^2^, which will gradually recover in 1 to 2 months. For larger exposed areas, the recommended salvage management is the application of temporoparietal fascia flaps (TPFs) and skin grafts.

### Infection

Infection occurs relatively more commonly in EH application cases and presents with unusual swelling, erythema, abscess and purulent secretion of the elevated auricle approximately 1 to 2 weeks or even several months after surgery. Conventional measures, such as timely drainage and irrigation, as well as intravenous antibiotics and hyperbaric oxygen therapy (HBOT), are normally adopted to treat infection. For uncontrolled infection in EH application cases, it is recommended to remove the artificial material.

### Exposure of support materials

The exposure of support materials mainly happens in EH application cases. Several reasons lead to their fracture or exposure, including a large area of facial flap necrosis, unstable fixation, long-term excessive friction or compression when sleeping and traumatic force. EH must be removed when exposed, and secondary surgical treatment should be considered.

### Hypertrophic scar

Hypertrophic scars will undoubtedly influence the final contour and symmetry of the cephaloauricular sulus. Intralesional steroid injection (Supplemental Fig. 1) and silicone sheeting application are the most commonly adopted methods to prevent and control hypertrophic scars^10^. In addition, an individualized three-dimensional printed ear splint can accommodate the auricle and exert appropriate pressure over the grafted skin, which is beneficial for forming a comparatively stable cephaloauricular sulcus (Supplemental Fig. 2)^11^. It is recommended to wear it initially 1 month after ear elevation when the skin graft has completely survived and continuously applied for 6 months even when sleeping or active.

## Results

We observed 895 microtia patients whose follow-up period ranged from 6 to 48 months. 78% of the patients were satisfied with the auricle contour with harmonious integrity. Twenty-nine patients developed partial skin necrosis and delayed healing. After effective conservative treatments, all wounds recovered gradually. With timely preventive measures, no obvious hypertrophic scars were observed, and the cephaloauricular sulcus was acceptable.

Thirteen patients experienced facial flap necrosis and cartilage exposure. Under conservative management, three cases healed completely with no further surgical treatment because of the limited exposure area, which was less than 10 mm^2^. For the other 10 patients with a comparatively larger exposure area but without exposure to support materials, TPF transfer and skin grafts were applied as salvage procedures. All essential subunits survived, and no further complications occurred.

Five patients experienced postoperative infections. After effective irrigation, aspiration, intravenous antibiotics and HBOT, three patients recovered completely. However, the other two cases presented repeated inflammation, and the artificial EH composite wedge had to be removed. Meanwhile, the exposure of the EH strut was found several months or years after ear elevation in another four cases. The EH wedge was subsequently removed, and secondary surgical treatment was needed thereafter.

Hypertrophic scars were found in 52 patients. The majority of them were in the early phase of this series. A sequence of therapeutic strategies, including steroid injection, silicone-based products, ear splints and surgical management, were effective to treat and prevent such complications. With the successful application of early intervention procedures, the amount of hypertrophic scarring has been controlled to a smaller range.

## Case reports

### Case 1

**Figure 2:**
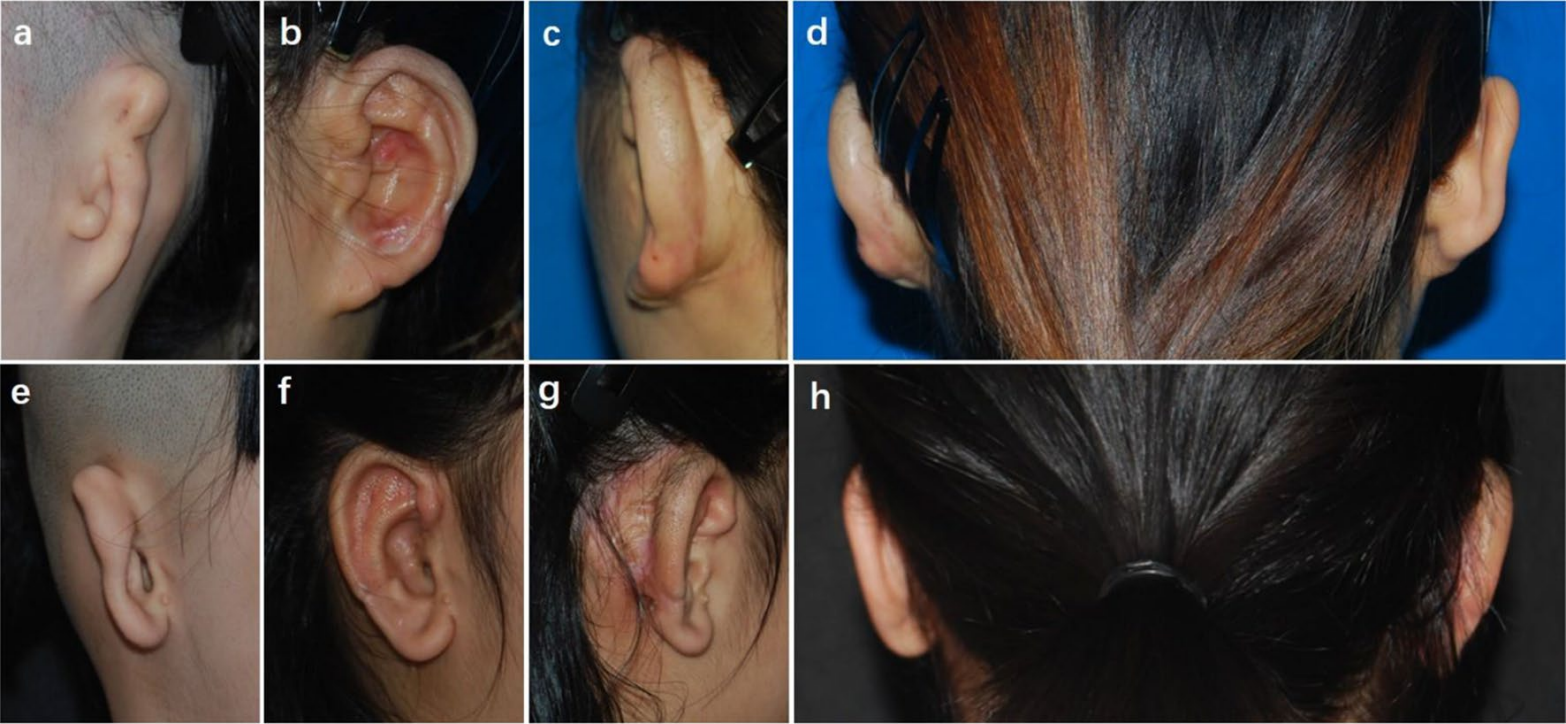
(**a**) A 26-year-old woman presented with congenital microtia, and she received ear elevation with autogenous cartilage as support material covered by RFF and a skin graft from the groin. (**b**) Postoperative oblique view at 30 months after ear elevation. (**c**) Postoperative oblique rear view showing that no obvious hypertrophic scar was observed. (**d**) Dorsal view showing that the cephaloauricular angle was nearly symmetric to the contralateral normal ear. (**e**) A 9-year-old girl presented with congenital microtia, and she received ear elevation with EH as support material covered by RFF and skin graft from scalp. (**f**) Postoperative oblique view at 12 months after the second stage of reconstruction. (**g**) Postoperative oblique rear view showing that no obvious hypertrophic scar occurred. (**h**) Dorsal view showing that the cephaloauricular sulcus approximated the normal side.

A 26-year-old woman presented with sausage-type microtia (Fig. 2a–d). A cartilage block was used as a support strut, which was covered by the superficial RFF and skin graft from the groin. It demonstrated a harmonious contour at 30 months after ear elevation. No obvious hypertrophic scar occurred, and the cephaloauricular angle was nearly symmetric to the contralateral normal ear.

### Case 2

A 9-year-old girl presented with concha-type microtia (Fig. 2e–h). An EH composite wedge plus the superficial RFF and skin graft from the scalp were applied in ear elevation. When followed at 12 months after the second stage, most essential morphologic features appeared in good definition. The cephaloauricular sulcus was satisfactory with no hypertrophic scarring.

### Case 3

**Figure 3:**
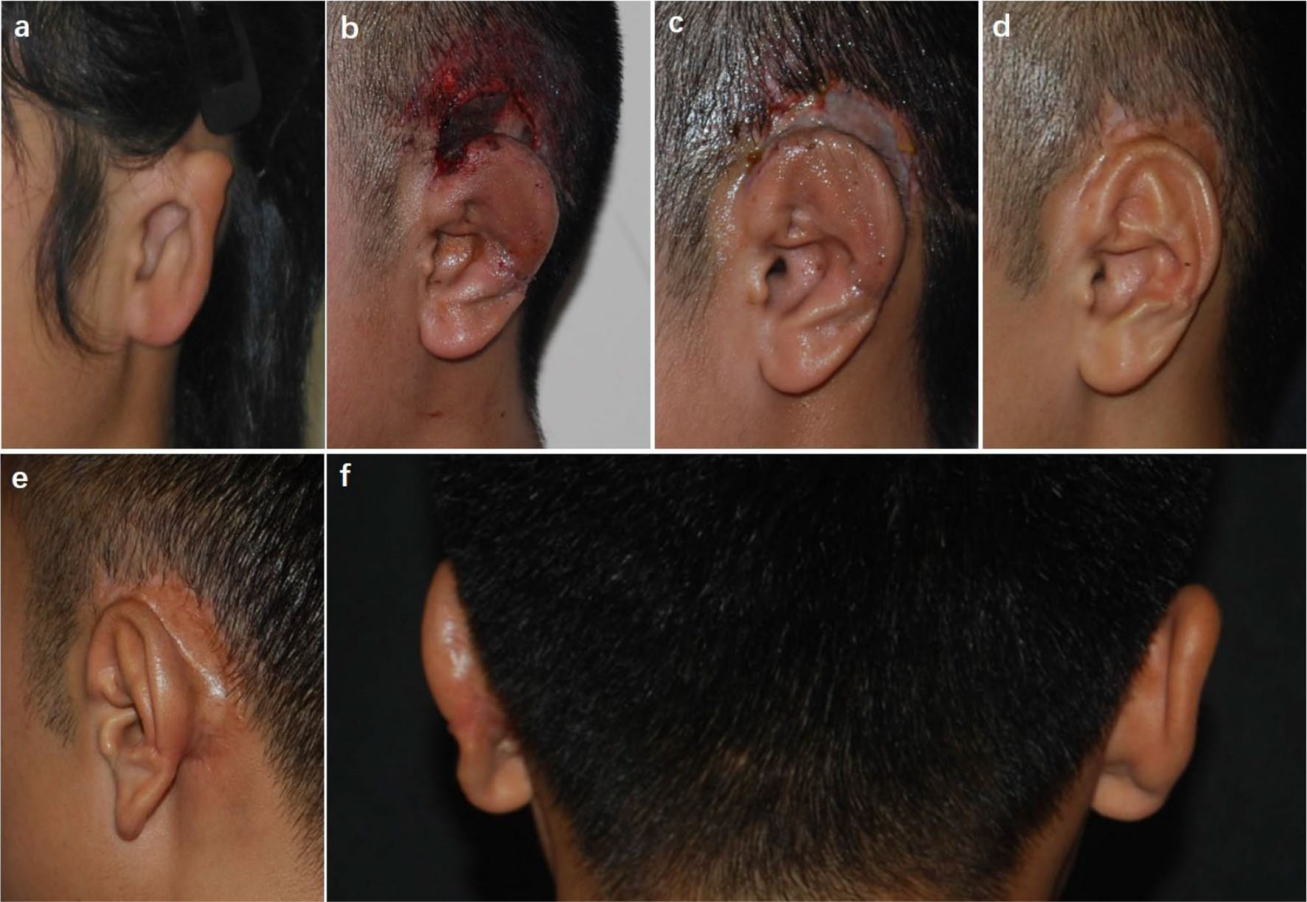
A patient with partial skin necrosis. (**a**) An 18-year-old patient presented with congenital microtia. (**b**) Partial skin necrosis was found at the dorsal margin of the auricle after the bolster was removed. (**c**) Delayed wound healing of the skin surface was observed after 20 days of HBOT. (**d**) Postoperative oblique view 19 months after ear elevation. (**e**) Postoperative oblique rear view showed that the cephaloauricular sulcus was smooth and that no hypertrophic scar was observed at the rear of the auricle. (**f**) Dorsal view demonstrated that the cephaloauricular angle approximated the normal ear.

An 18-year-old patient suffered from partial skin necrosis at the rear of the auricle after the bolster was removed (Fig. 3). Delayed wound healing of the skin surface was observed after 20 days of HBOT, and further relevant anti-scar procedures were applied. No significant hyperplastic scars were found at the rear of the auricle, and the auricle contour was satisfactory 19 months after ear elevation.

### Case 4

**Figure 4:**
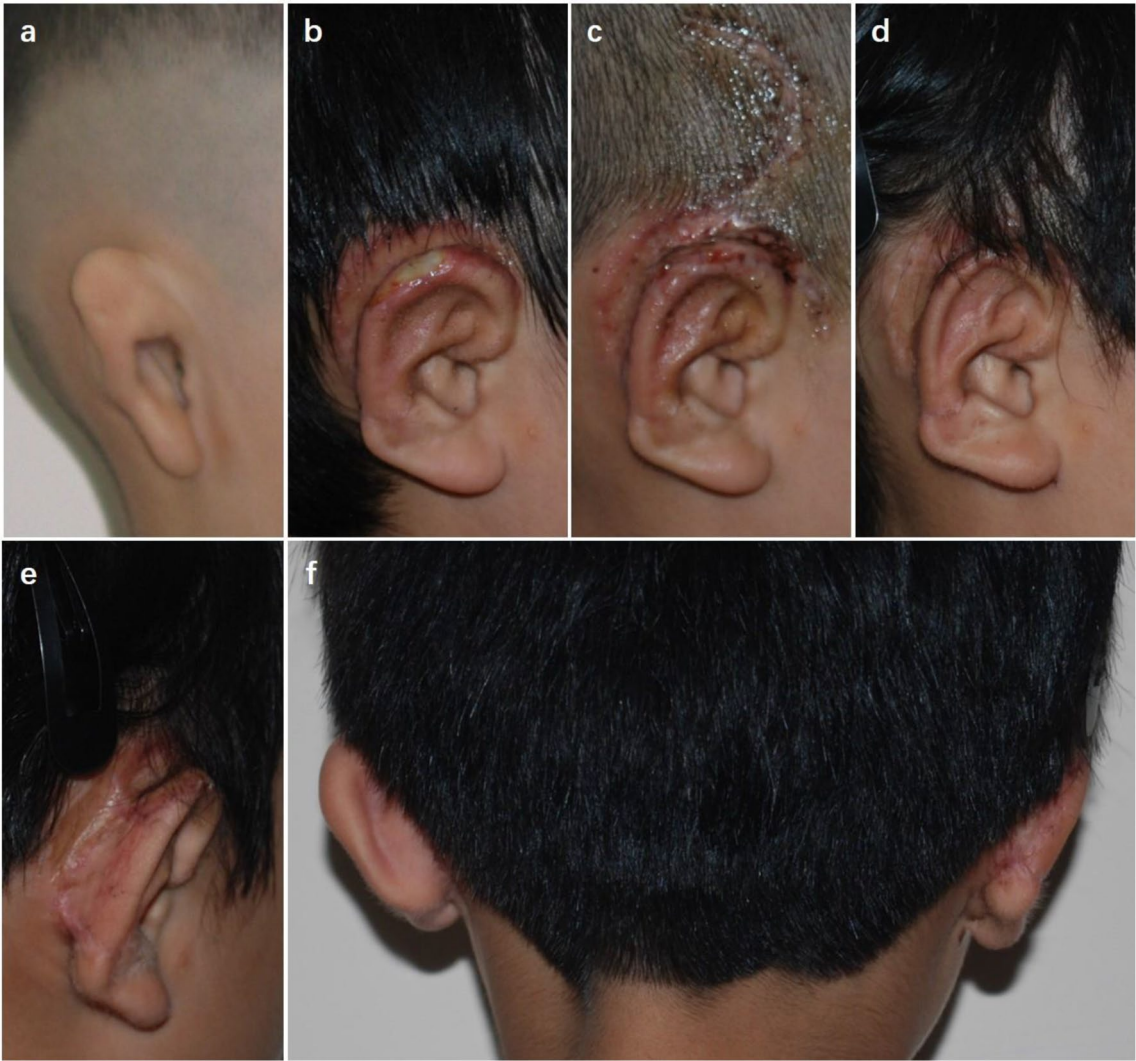
A patient with fascial necrosis and cartilage exposure at the dorsal part of the helix. (**a**) An 8-year-old boy presented with congenital microtia. (**b**) Cartilage exposure of approximately 2 cm^2^ at the superior dorsal part of the helix was found 3 weeks after surgery. (**c**) Two weeks after the salvage operation with TPF and skin graft. (**d**) Postoperative oblique view 6 months after the repair operation. (**e**) Postoperative oblique rear view showing that the cephaloauricular sulcus was deep and no obvious hypertrophic scars were observed. (**f**) Dorsal view showing that the cephaloauricular angle was acceptable.

Three weeks after surgery, the necrosis of the fascial flap and cartilage exposure were found at the dorsal part of the helix in an 8-year-old boy patient (Fig. 4). He underwent a salvage operation to cover the wound with TPF and a skin graft. The cephaloauricular angle approximated the normal ear, and the auricle contour was acceptable at follow-up 6 months after surgery.

### Case 5

**Figure 5:**
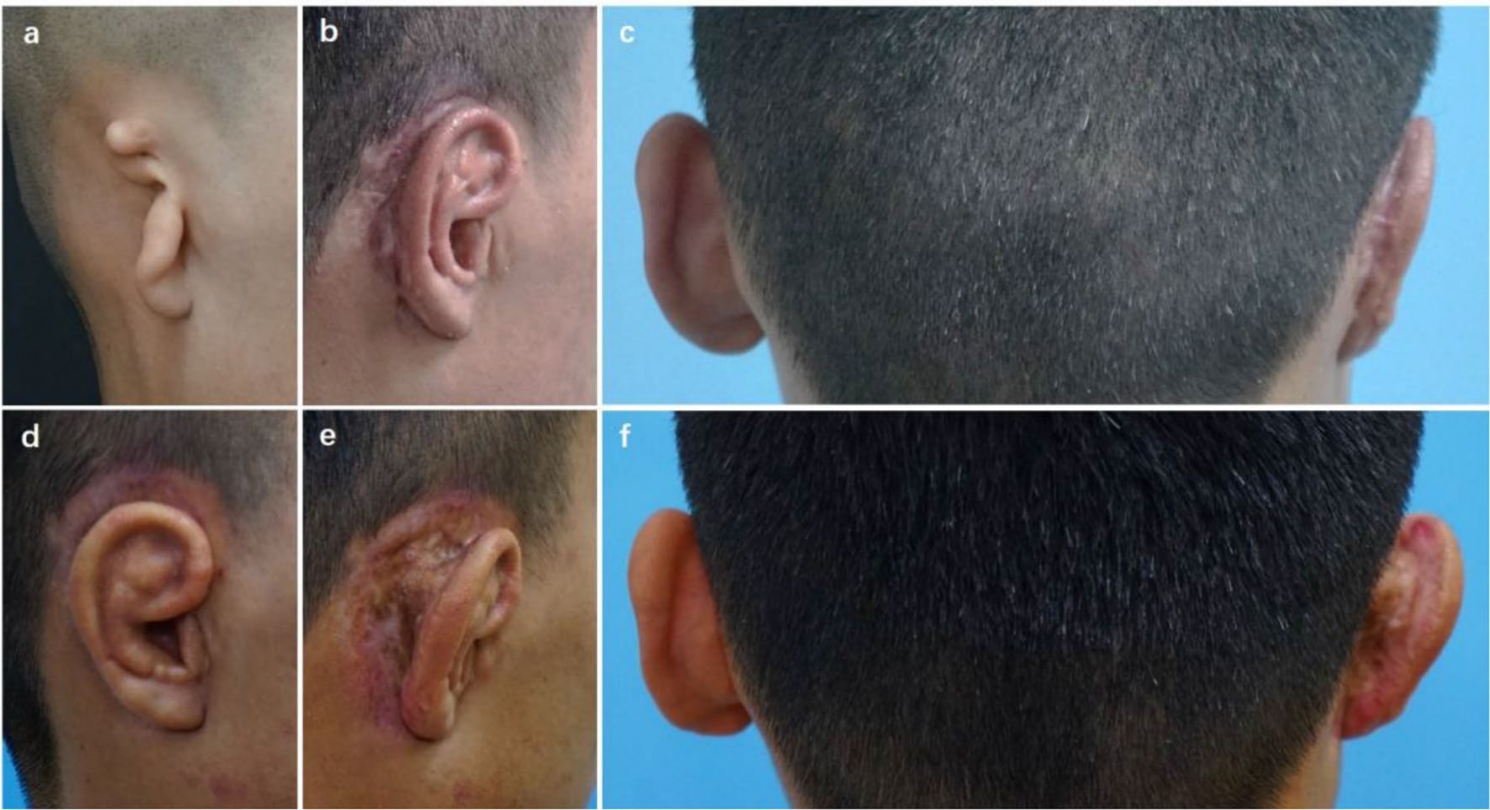
A patient with hypertrophic scars and contraction of the cephaloauricular angle. (**a**) A 19-year-old man presented with congenital microtia, and he received ear elevation with autogenous cartilage as support material covered by RFF and a skin graft from the scalp. (**b**) Hypertrophic scars were found 6 months after ear elevation. (**c**) *C*ontraction of the cephaloauricular angle was noted simultaneously. (**d**) Postoperative oblique view at 6 months after secondary surgery, in which the scars were excised and an additional cartilage block was harvested as support strut covered by TPF and skin graft from scalp. (**e**) Postoperative oblique rear view showing that the cephaloauricular sulcus was deep and that no hypertrophic scars were found. (**f**) Dorsal view demonstrated that the cephaloauricular angle was nearly symmetric to the contralateral normal side.

Hypertrophic scars and contraction of the cephaloauricular angle occurred in a 19-year-old male patient 6 months after ear elevation (Fig. 5). For secondary surgery, the scars were excised, and an additional cartilage block was harvested as a support strut, which was then covered by TPF and skin graft from the scalp. With the application of early intervention management, the cephaloauricular sulcus was deep, and the cephaloauricular angle was nearly symmetric to the contralateral normal side at the 6-month follow-up after the salvage operation.

## Discussion

For staged autologous cartilage microtia reconstruction, the aim of ear elevation is to attain a deep and stable cephaloauricular sulcus with proper projection^12^. However, the surgical procedures are changeable and undetermined. Moreover, various complications, especially hypertrophic scarring, undoubtedly impact the posterior appearance of the auricle. Therefore, it is essential to develop a practical project and proper strategies for the prevention and treatment of postsurgical complications.

The space and angle of the normal cephaloauricular sulcus become gradually wider and projective from the top one-third to the middle and lower portions^13^. Given the comparatively narrow space with a sharp angle in the upper third region and principal soft tissue at the base of the caudal region, cephaloauricular struts are commonly placed at the middle third cephalic area just beneath the central part of the antihelix, which can provide strong and rigid base support to the materials on top. A reliable upholder is a prerequisite for the successful construction of functional and aesthetic cephaloauricular sulci. Autogenous costal cartilage has been widely accepted as a support material because of its excellent biocompatibility and few complications. To provide a firm elevation of the constructed auricle and resist contraction force from the grafted skin, the cartilage strut should have adequate volume. Specifically, dimensions of 12–15 mm in height, 20 mm or more in length and no less than 4–5 mm in thickness are fundamental to attain a symmetric projection. If the residual cartilage from the first stage could not match the minimum requirements, particularly in some children, artificial materials such as porous polyethylene^14^, MEDPOR^15^, EH^16^ and titanium mesh strut^17^ could be an alternative option.

The original EH composite we used, as described in our previous report^16^, is 10 mm in height at the upper and lower extremities and 8 mm in the middle part. Given the comparatively large volume, it needs to cover a wider area of the fascia flap and simultaneously increases the risk of exposure. Thus, we modified the long EH composite wedge with projected two ends to a “C” shape with reduced length and volume. By adopting this reformation, it could stand firmly on the right posterior wall of the conchae in the middle third part of the ear. Moreover, the shape lessened the risk of exposure resulting from long-term compression when sleeping, particularly at the top of the two ends. In addition, it lowered the dimension and burden of the blood supply of the covering fascia flap.

TPF and RFF are two common options of coverage for the inner strut and posterior part of article^3^. An axial pattern of TPF provides a thinner fascial flap with reliable blood supply. However, the safe elevation of the TPF is a time-consuming procedure, and alopecia along the long incision line may be obvious in male patients with a short hair style. Meanwhile, the pedicle at the upper pole of the auricle usually demonstrates a shallower and obtuser sulcus^12^. Therefore, we recommend keeping the TPF for a secondary salvage procedure. In this series, we applied RFF because of easy and timesaving handling with shorter incision lines and fewer conspicuous scars. Moreover, referring to anatomical studies, the upper portion of RFF generally originates from the superficial TPF, which demonstrates more advantages, such as a thinner, more elastic and highly vascularized histological appearance^18^. In contrast, the lower region is mainly from the superficial mastoid fascia, which is thicker, heavier and not distinctly separated^19^. Thus, we recommend harvesting the upper portion of RFF rather than the caudal part.

Split-thickness skin grafts from scalps or full-thickness grafts from the ilioinguinal region are most commonly used to cover skin defects at the rear of the auricle. The groin skin graft has a lower degree of shrinkage than that from the scalp, but poor color match and inconvenience from immobilization at the early recovery stage affect the acceptance of patients^20^. In comparison, skin grafts from the scalp are accepted more frequently because of favorable color matches, concealed scars and no limit in early postoperative motion. Although skin shrinkage and scar hyperplasia occur more commonly, they can be controlled and ameliorated by early intervention, such as the prophylactic injection of triamcinolone, the application of silicone-based products and individualized ear splints. Hence, the scalp is still the premier option of most patients and plastic surgeons as the donor site of skin grafts^21^. Other skin donor regions, including the chest, inner brachia and abdomen, are seldom chosen on account of additional obvious scarring.

When partial skin necrosis occurs, which does not implicate a fascia flap or cartilage, conventional therapeutic procedures such as the local application of antibiotic-impregnated ointment, regular dressing change and HBOT are effective, and the wound can recover by delayed healing. There is evidence in randomized studies that HBOT accelerates wound healing compared with protocols without HBOT^22,23^. To improve the survival ratio of the fascial flap and skin flap, we recommend the posterosuperior auricular fascia flap (PSFF), which is thinner and easier for dissection and can be harvested in sufficient size with stable blood circulation. In addition, it is vital to implement meticulous hemostasis in the surgery and ensure adequate drainage of skin grafts as well as appropriate and evenly distributed bolster pressure.

If facial flap necrosis occurs and the region of cartilage exposure is less than 10 mm^2^, conservative therapy approaches are practicable. TPF and skin grafts are normally considered to salvage larger areas of exposure. Given the potential threat to the final contour and cephaloauricular sulcus of the auricle, we think it crucial to implement practicable strategies to prevent the occurrence of such complications. First, a smooth rear of the base frame should be obligatorily ensured during cartilage fabrication at the first stage. Meanwhile, any palpable sharp end of protruded steel wires at the dorsal part should also be removed when the framework is elevated at the second stage. Moreover, the ends of the steel wires were deeply buried untouchably after the support materials were fixed. Otherwise, such sharp protrusion of cartilage or steel wires would definitely decrease the survival area of the fascial flap. In addition, harvesting PSFF with a reliable vascular supply, stable fixation of the fascial flap by interrupted sutures and proper pressure of bolster compression are also important to lessen the risk of facial flap necrosis and subsequent cartilage exposure.

Infection, despite its low occurrence rate, has a fatal effect on implanted support materials, especially EH composites, and thereby influences the symmetry of the cephaloauricular angle. Thus, it is of great importance to take preventive sterile measures and prophylactic antibiotics in the perioperative period. In general, a combination of sensitive intravenous antibiotics, sufficient drainage and timely irrigation, as well as HBOT^24^, are effective to treat infection. However, the presence of recurrent infections in EH application cases may finally result in material removal.

Hypertrophic scarring manifests abnormal organization of collagen at the remodeling phase of wound healing^25,26^, which may influence the final auricular contour and symmetry of the cephaloauricular angle. Tension and wound complications are two main risk factors for scarring. Therefore, the fascia flap should have sufficient size with reliable blood supply and accommodate the dorsal wound region with no tension. Meanwhile, the fine eversion of the skin edges evenly distributes tensile strength at each side and thus efficiently avoids wound dehiscence^27^. Moreover, strict sterile measures, prophylactic antibiotics and precautious wound care can absolutely minimize inflammation and infection.

The intralesional injection of triamcinolone acetonide (TAC) has been proven effective and commonly applied as a first-line approach for the prevention and treatment of hypertrophic scars, whose mechanism involves inflammation inhibition, fibroblast growth reduction, collagen synthesis suppression and collagen degeneration promotion^28^. Prophylactic injection can initiate at the mastoid area and tends to exhibit signs of hypertrophic scarring, such as mild redness or unevenness. It can also be applied in combination with 5-fluorouracil (5-FU) in adult patients, which can reduce the dosage and lessen side effects of steroids, such as dermal atrophy and depigmentation^29^.

Silicone-based products are also preferred because of their noninvasiveness and accessibility^30^, whose mechanism is to regain the water barrier through the occlusion and hydration of the stratum corneum and thereby contribute to limiting excessive scarring^31^. We found that ear splints also play an important role in maintaining the stability and depth of the cephaloauricular sulcus. Pressure between 24 and 30 mmHg can achieve maximum function because it will effectively reduce the synthesis of collagen and diminish hypertrophic processes by blocking capillary blood supply but not influence peripheral blood circulation^32^. The key points are long-term wear under supervision and timely follow-up for possible adjustment. Despite various preventive measures introduced, some patients may still develop hypertrophic scars or keloids due to questionable compliance, untimely follow-up or special physical constitution of patients. Necessary surgical excision combined with intensified adjuvant approaches would be essential to control the recurrence of severe scars.

Auricular reconstruction with skin dilators is another optional technique, especially in hemifacial microsomia patients with a low hairline and taut postauricular skin. This method could not only provide sufficient skin coverage but also achieve color and texture matching as well as sensory preservation. Significant improvements have been made in tissue expansion to minimize skin grafting^4,33–36^. However, a long expansion duration and multiple operations are still necessary to obtain adequate expanded skin. The dilemma of treating complications such as infection, skin necrosis and tissue expander exposure has limited its popularization. In addition, more cartilage would be sacrificed to strengthen the framework in order to resist the inevitable and unpredictable contraction of the skin flap. Therefore, the successful application of tissue expanders in microtia reconstruction relies on extensive experience and the appropriate selection of indications. In comparison, the two-stage method modified from Brent and Nagata’s technique we applied in this series is time-saving, easy to learn and widely popular. Comparatively less cartilage is needed to strengthen the framework because of mild and predicable skin flap contraction. Therefore, this approach lessens the occurrence of chest deformities and results in a reconstructed auricle with a more harmonious contour. Additionally, this method involves fewer hospitalizations and complications, thereby facilitating postoperative activity and recovery, and is thus more acceptable to surgeons and patients.

## Conclusions

Individualized strategies for ear elevation and complication treatment in autogenous cartilage microtia reconstruction contribute to achieving a satisfactory projection and contour of the reconstructed auricle.

## Supplementary Information

Supplementary Figures.

## Data Availability

The datasets used and/or analysed during the current study available from the corresponding author on reasonable request.
