# Supplementary material for: Strategies for ear elevation and the treatment of relevant complications in autologous cartilage microtia reconstruction
Source: Sci Rep. 2022 Aug 8;12:13536. doi: 10.1038/s41598-022-17007-3 (PMC9360043; doi:10.1038/s41598-022-17007-3)
Supplement: Supplementary file 1 — Supplementary Figures. [file 41598_2022_17007_MOESM1_ESM.pdf]

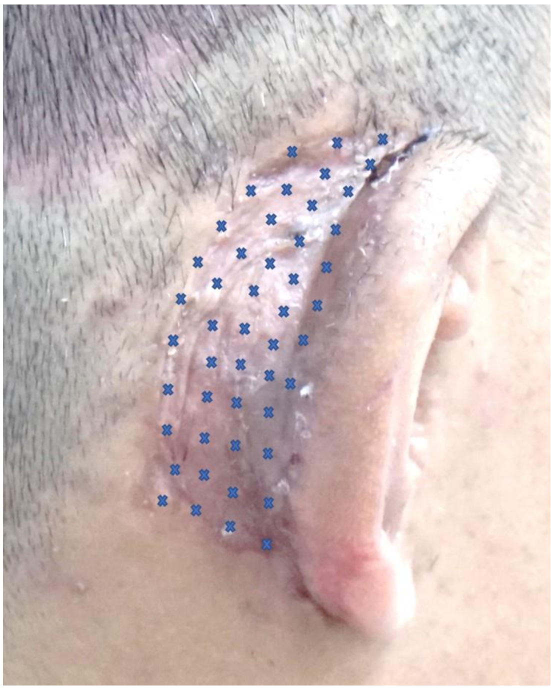

Supplemental Fig.1. Schematic of prophylactic intralesional steroid injection. Triamcinolone acetonide 4.0 mg/ml diluted in 2% lidocaine in the same volume ratio was injected as 0.1 ml each point by a 1 ml syringe with a 29-gauge hypodermic needle at 5 mm intervals mainly at the mastoid region of grafted skin when the wound healed completely approximately 3 weeks after ear elevation. The maximum dose each time was no more than 40 mg. The blue marks show the injection points.

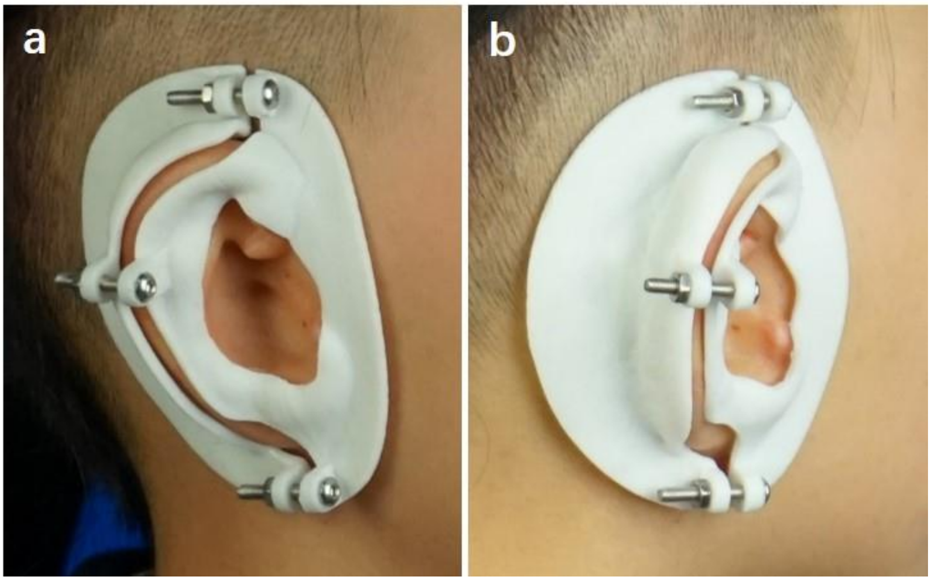


Supplemental Fig.2. Schematic of an individualized three-dimensional printed ear splint. *(Left)* The anterior and posterior pieces were fixed with three screws during application. The tightness of the brace could be adjusted using the three screws in accordance with the patient's comfort. *(Right)* Lateral view of the ear splint.
